# Understanding unequal ageing: towards a synthesis of intersectionality and life course analyses

**DOI:** 10.1007/s10433-020-00582-7

**Published:** 2020-10-15

**Authors:** Daniel Holman, Alan Walker

**Affiliations:** Department of Sociological Studies, The University of Sheffield, Elmfield, Northumberland Road, Sheffield, S10 2TU UK

**Keywords:** Intersectionality, Life course, Cumulative disadvantage, Unequal ageing, Health inequalities

## Abstract

Intersectionality has received an increasing amount of attention in health inequalities research in recent years. It suggests that treating social characteristics separately—mainly age, gender, ethnicity, and socio-economic position—does not match the reality that people simultaneously embody multiple characteristics and are therefore potentially subject to multiple forms of discrimination. Yet the intersectionality literature has paid very little attention to the nature of ageing or the life course, and gerontology has rarely incorporated insights from intersectionality. In this paper, we aim to illustrate how intersectionality might be synthesised with a life course perspective to deliver novel insights into unequal ageing, especially with respect to health. First we provide an overview of how intersectionality can be used in research on inequality, focusing on intersectional subgroups, discrimination, categorisation, and individual heterogeneity. We cover two key approaches—the use of interaction terms in conventional models and multilevel models which are particularly focussed on granular subgroup differences. In advancing a conceptual dialogue with the life course perspective, we discuss the concepts of roles, life stages, transitions, age/cohort, cumulative disadvantage/advantage, and trajectories. We conclude that the synergies between intersectionality and the life course hold exciting opportunities to bring new insights to unequal ageing and its attendant health inequalities.

## Introduction

The main aim of this paper is to illustrate how intersectionality might be synthesised with a life course perspective to deliver new insights into unequal ageing, especially with respect to health. First we outline the background to intersectionality and its potential for furthering knowledge on health inequalities, noting the potential for mutual enrichment between intersectionality and gerontology. We then discuss the key concepts and debates in intersectionality before considering the approaches that are typically used and a recently developed multilevel method. Finally, we provide a brief account of the life course perspective before considering how its synthesis with intersectionality can help to further understanding of unequal ageing. Our secondary aim is to encourage further analyses of unequal ageing from an intersectional life course perspective. This paper focuses on quantitative methods, but also draws on the wider literature for illustrative purposes.

## Background

Intersectionality originated some thirty years ago in the USA and has subsequently attracted wide interest. Crenshaw (1989: 155) coined the term to describe how the experiences of Black women in the legal system were shaped by ‘crosscurrents of racism and sexism’. She used the example of an employment discrimination lawsuit filed by five Black women against General Motors to illustrate the idea. The company hired both women and Black people, but had a poor record of hiring Black women in particular. The court rejected the discrimination case based on this ‘special sub-category’, arguing that ‘Black woman’ is not a legally protected class under civil rights law and that ‘the prospect of the creation of new classes of protected minorities, governed only by the mathematical principles of permutation and combination, clearly raises the prospect of opening the hackneyed Pandora’s box’ (Crenshaw 1989: 142). Crenshaw’s motivation for outlining intersectionality was that without a way to frame such subgroup inequalities they can be rendered invisible; focussing on only one social characteristic at a time is a ‘trickle down approach to social justice’ (Crenshaw 2016). Thus, the main value of intersectionality is that it directs attention towards subgroups that face disadvantages which might otherwise go undetected.

Recently, intersectionality has been suggested as a promising way to advance health inequalities research precisely because it is well-suited to addressing and elucidating diverse inequalities (Bowleg 2012) and how they are driven by multifaceted power structures and processes (Kapilashrami and Hankivsky 2018)—especially systems of discrimination such as sexism and racism that characterise social hierarchies. Discrimination—the unjust, unfair treatment on the basis of social categories—has been shown to be an important determinant of health inequalities (Krieger 2014). Given its focus on diverse inequalities and the processes that drive them, intersectionality has also been seen as a rich framework for equity-driven health policy analysis (Hankivsky et al. 2014). It has been argued that the knowledge generated by intersectional analyses could provide evidence for implementing ‘proportionate universalism’ (Merlo et al. 2019), the idea that health services and actions should be resourced and delivered with a scale and intensity proportionate to the level of disadvantage (Marmot et al. 2020). Highlighting particularly vulnerable subgroups might suggest targeted policies (Lofters and O’Campo 2012), although categorisation and heterogeneity are essential to consider in such an approach, as we explore below. A recent series of papers in *Social Science & Medicine* have focussed on intersectionality (Bauer 2014; Bauer and Scheim 2019; Evans et al. 2018; Gkiouleka et al. 2018; Green et al. 2017; Lizotte et al. 2019; Merlo 2018; Warner and Brown 2011), and a paper in *The Lancet* argued that it is important for global public health and the ‘leave no one behind’ agenda (Kapilashrami and Hankivsky 2018).

Yet the intersectionality literature has paid very little attention to the nature of ageing or the life course, and gerontology has rarely incorporated insights from intersectionality (Calasanti and King 2015; Koehn et al. 2013). As Corna (2013: 154) notes for example with respect to gender, ‘current scholarship may be overlooking how gender and social policy contexts intersect to influence socio-economic inequalities, and their relationship to health, differently for men and women over time’, despite gender being a fundamental axis of inequality in education, the labour market, the family, and later life pension wealth. Similarly, Dannefer (2018) argues that instead of controlling for race, socio-economic position (SEP), and gender, as is typically done in life course research, we should embrace a more complex systems and holistic view of how they might interact.

Bringing together intersectionality with ageing research was proposed in an earlier paper by Dressel et al. (1997), which from a gerontological perspective argued that ideas around intersectionality and interlocking oppressions can be used to better understand how structural factors shape ageing. Ferrer et al. (2017) argued for combining concepts from intersectionality and life course from an ethnographic perspective. We discuss this existing work and provide empirical examples in more detail below.

## Intersectionality: key concepts and debates

### Intersectional subgroups

Intersectionality reflects the fact that people embody multiple social characteristics such as gender, ethnicity, age, socio-economic position (SEP) simultaneously. Combinations of social characteristics constitute different potential intersectional subgroups, for example a working-class 55-year-old Black woman. Intersectional subgroups represent (1) (objective) positions in the social hierarchy and (2) (subjective) social identities (Bauer 2014). They are also therefore associated with differential power/resources, life chances, and lived experiences. Further, and fundamentally for intersectionality, because subgroups are (at least partly) socially distinctive and relational (i.e. ‘female’ is defined in contrast to ‘male’), they make discrimination possible. For example, a working-class 55-year-old Black woman typically has distinct power/resources, life chances, and lived experiences to a 25-year-old White middle-class man and is more likely to be discriminated against.

Despite intersectional patterning, it is important to avoid deterministic understanding and instead acknowledge how agency mediates intersectional effects (Nash 2008). For example, alongside considering how discrimination impacts on health, we should also consider ‘how people resist injustice and its health-harming effects, individually and collectively, and the resilience that enables them to do so’ (Krieger 2014: 656). Structural position and social identity are therefore not always in accordance with each other, as people can resist social categories or be empowered to engage with them in different ways. Opportunities to do so are to some extent context dependent (e.g. with respect to place and time) (Bauer 2014). Much work is yet to be done to understand how intersecting identities are experienced and in what circumstances and contexts. Corlett and Mavin (2014) have usefully set out a future research agenda on this topic.

### Interlocking systems of discrimination: the ‘matrix of domination’

Discrimination is rooted in the relationships of dominance and oppression that characterise social hierarchies. Stereotypes are central to this, which can be either descriptive in terms of what a social group are perceived to be like, e.g. older adults being stereotyped as being weak, sick, or senile (Palmore 2015) or prescriptive, e.g. older adults are expected to not consume too many resources or engage in activities seen as traditionally for ‘younger’ people (Centre for Ageing Better 2020). Discrimination can be either interpersonal (perceived discrimination in face-to-face encounters), institutional (policies or practices of state or non-state institutions), or societal (the totality of ways in which societies foster discrimination, e.g. in housing, education, employment, earnings, social security) (Krieger 2014). Institutions are important for understanding unequal ageing as they not only have stratifying effects on health, but also ‘open possibilities for social connections and collective action’ (Gkiouleka et al. 2018). Typically, researchers have focussed on welfare states, but there is a need to consider the health effects of other types of institutions, such as education, employment, and incarceration, and to consider how these effects interplay with individual positioning through multiple axes of power. Corna (2013) provides a framework for how researchers can examine the links between institutional policies and health which we describe in more detail further below.

Systems of discrimination such as sexism, racism, ageism, and classism are multiple and ‘interlocking’: the ‘matrix of domination’ according to Collins (2002: 228), ‘within which intersecting oppressions originate, develop, and are contained’. She elaborates that ‘In the USA, such domination has occurred through schools, housing, employment, government, and other social institutions that regulate the actual patterns of intersecting oppressions that Black women encounter’. The ‘matrix of domination’ therefore describes a society’s interconnected configuration of sexism, racism, ageism, classism, and other types of discrimination, as rooted in its institutions, policies, practices, as well as in face-to-face encounters. An example of how systems of discrimination overlap is evident in intersectional stereotypes. Against a prejudiced backdrop of Black Americans as lazy and unintelligent, Black women in particular are often viewed as welfare ‘mammies, matriarchs, and welfare recipients’, while black men are stereotyped as criminals and rapists (Krieger 2014: 651). Gerontologists have considered the interplay between ageism and other forms of discrimination, most notably with respect to sexism (e.g. Krekula 2007). Yet empirical studies are rare. A recent systematic review of individual and structural ageism including over 7 million people and 422 studies around the world confirmed its pernicious health effects yet noted that only 18 studies had analysed interactions with other forms of discrimination, leading the authors to argue that intersectionality is an important area of future research on this topic (Chang et al. 2020).

### Categorisation

Although intersectionality was originally concerned with the position/identity of Black women (and crosscurrents of racism and sexism), this focus ignores how Black women are differentiated according to other axes of inequality, such as social class or nationality (Nash 2008). Poor Black women have different experiences in housing, education, and employment compared with wealthy Black women. This does not mean that studies on ‘Black women’—such the seminal contributions of Crenshaw (1989) and Collins (2002)—lack value; the experience of being a Black woman to some extent transcends socio-economic lines. Similarly, studies on single categories of difference and discrimination, including age/ageism or gender/sexism, have provided valuable contributions on fundamental dimensions of difference and inequality. Yet, if we do not consider how such fundamental dimensions intersect, we assume that the experience of all women or people of a certain age for example are the same and will likely miss important subgroup inequalities.

Given the wide availability of data on gender, ethnicity, age, and socio-economic position and that these are known to be key dimensions of inequality, a sensible default position for intersectionality research is to include them. Other axes of inequality such as nationality, disability, and sexuality should be included if relevant to the topic and the data allow. Yet they typically receive far less attention—a reflection of dominant groups often deciding which other groups will be the subject of study, and how they will be studied (Dressel et al. 1997). The increasing availability of ‘big data’ in the form of linked administrative data and biobanks, for example, suggests that there are new untapped opportunities for intersectional life course research. However, the recording of social variables is often sketchy in such data, and ideally investment in rich large-scale prospective cohort studies is needed to fully capture of the experience of marginalised groups, especially. A further decision for researchers to take is how each axis of inequality is categorised. Intersectionality prompts us to question traditional categorisations. For example, measuring ethnicity using a White/Non-White indicator might be the only option given data availability constraints but nonetheless misses important variations in the population (Gkiouleka et al. 2018). Gender is often taken-for-granted as binary, though to properly capture diversity a non-binary category might also be included. Socio-economic position has many potential measures such as income, occupation, and wealth, which are more or less relevant at different points in the life cycle. We discuss these life course dynamics further below.

Once axes of inequality have been included and categorised, researchers need to decide which intersectional positions/locations resulting from combinations of these categories they should focus on. As noted by Bauer (2014), few people are disadvantaged according to all axes of inequality; rather, people typically experience of a mixture of advantage and disadvantage (though some much more than others). From a health inequalities perspective, all intersectional positions/identities are potentially of interest (Aisenbrey and Fasang 2018; McCall 2005), including more powerful and privileged ones in order to understand advantage, and marginalised and oppressed ones to understand disadvantage, and identify both protective and risk factors—highly relevant for social equity policies. This suggests an exploratory approach in the sense of which particular intersections to focus on can be justified. Replication in such an approach is of course crucial.

Supposing complete population data are available (e.g. as with register data) with rich measures, researchers might be tempted to include a range of axes of inequality and categories within each to capture the ‘true’ complexity of intersectional patterning in the population. It is important to note however that it is not the purpose of intersectionality to define ever more risky granular subgroups (Green et al. 2017). Further, it soon becomes apparent that adding enough granular detail according to how intersections are defined will lead to conclusion that ‘everyone is different’—a unique combination of social characteristics. As more and more granularity is added, categorisation would eventually result in each intersection containing only one person, and the commonalities between people are lost. This is the ‘infinite regress’ of sub-categorisation (Davis 2008) and was a key reason why the court rejected the discrimination case Crenshaw referred to. Further, complexity can lead to ‘deficit thinking’, by focussing on particularly disadvantaged intersections, and not on their assets and resilience. This can mean that ‘power relations and material inequalities that constitute oppression remain obfuscated’ (Dressel et al. 1997: 581). In contrast to a broad approach mapping disadvantage/advantage across multiple axes and categories of inequality, a more theoretically informed approach might focus on specific intersectional positions/identities (Bauer 2014). As noted, intersectionality theory itself has now moved away from an exclusive focus on Black women and has instead incorporated multiple axes of inequality and their resulting intersections. Beyond intersectionality, particular theories when considered together, such as feminism and Marxism, might suggest prioritising a focus on, for example, working-class women. In our view, both exploratory and deductive approaches to selecting intersectional subgroups for study are justified for the reasons stated. It is also important to note that not all inequalities research or policy should be intersectional: focussing only on for example gender or age inequality can be a type of ‘strategic essentialism’—a way to stress commonality over difference to help mobilise action (Smith 2016).

### Individual heterogeneity

Even within tightly defined intersectional positions substantial individual heterogeneity will always remain; in other words, not all those who are, e.g. a working-class 55-year-old Black woman are the same, in terms of health, or otherwise. This ecological fallacy can risk stereotyping and stigmatising particular subgroups (Merlo 2018) and amounts to essentialism in regarding social categories as immutable properties of individuals rather than socially constructed (Dressel et al. 1997). Suggestions to target policies based on intersectional differences need to take this into account, which might not only lead to stigma, but might also be inefficient. Indeed, recent empirical findings suggest that, in fact, intersectional subgroups exhibit a high degree of overlap in terms of their distribution with respect to a range of health outcomes (Evans and Erickson 2019; Fisk et al. 2018; Hernández-Yumar et al. 2018; Persmark et al. 2019), which might also be expected with regard to other subgroup characteristics. Thus, intersectional patterning illustrates the diversity of population inequalities, but belonging to an intersectional subgroup does not define individual experience, not least because of the role of human agency. Nonetheless, average intersectional differences might be useful from a policy perspective because they might suggest that societal (and therefore modifiable) factors underlie the population distribution of individual risk (Merlo 2018).

## Intersectional approaches and methods for health inequalities research

### Inter- and intracategorical approaches to intersectionality

Once categories have been defined, two main approaches to intersectionality relevant to inequalities research can be distinguished: inter- and intracategorical (McCall 2005). The first is concerned with differences between intersections, the second with focussing within particular intersections without necessarily comparing them with others. Qualitative methods are especially suited to this approach since they are able to explore the complexities and diversity of the social lives of those who occupy different intersections (McCall 2005) (a fundamental aspect of which is time and timing). In a recent guide to how researchers can apply intersectionality theory to qualitative health research, Abrams et al. (2020) suggest that it should be deployed from a critical perspective, but do not go on to prioritise a particular theoretical framework, arguing instead that the approach to be taken depends on the aim of the research. Scholars in the intracategorical tradition tend to focus on marginalised intersections rather than dominant ones (McCall 2005). However, not all intersectionality scholars advocate for categorisation. Anti-categorical scholars reject categories outright, arguing that they are false social constructions and unhelpful simplifications of the complexity of the social. Yet for quantitative research it is necessary to accept categories to some extent, even if acknowledged as socially constructed, somewhat imperfect, and always in flux (McCall 2005).

### Analysing intercategorical intersectionality using interaction terms

Quantitative analyses have typically used interaction terms to analyse intercategorical intersectionality. *Not* specifying interaction terms can lead to false conclusions in examining subgroup differences. Bowleg and Bauer (2016) give an example by Schulman et al. (1999) who in their study of gender and race bias in cardiac catheterisation specified only main effects and did not include an interaction. They concluded that men and Whites were more likely to be referred for catheterisation than women and Blacks. In fact, correctly including the interaction term, Black women in particular were the *only* group with lower odds of referral. Interaction terms can be specified in nearly all types of analysis, including in longitudinal approaches, though caution must be exercised because their interpretation depends on whether the model is linear (e.g. linear regression) or not (e.g. logistic regression). Alternatives to specifying interaction terms to study intersectionality are also sometimes used—see Bauer (2014) for an excellent review of this topic.

### Additive versus multiplicative effects

Analysing interaction terms tests for multiplicative interaction (in linear models). Yet two types of statistical effect drive intersectional subgroup differences: additive or multiplicative (or some combination thereof). Additive effects represent the layering of disadvantage/advantage, whilst multiplicative effects might suggest that the effect of one social characteristic is attenuated or amplified by another. For example, with only additive effects, the effect of ethnic minority status on health is the same regardless of gender, and with multiplicative effects, the effect of ethnic minority status on health might be particularly pronounced for women. This begs the question of whether the absence of an interaction effect, i.e. where no statistical multiplicativity is present, somehow disproves intersectionality. As noted by Bauer and Scheim (2019), some scholars have erroneously assumed that intersectionality only refers to multiplicative effects, and their absence falsifies it. Rather than a falsifiable hypothesis, however, intersectionality is above all a framework for understanding heterogeneity and social power (Bauer and Scheim 2019; Bowleg 2008). Emerging research suggests that there are typically large differences in health between intersectional subgroups, e.g. the health of a working-class 55-year-old Black woman is typically worse than that of a 25-year-old White middle-class man, and these are mostly driven by additive rather than multiplicative effects (Holman et al. 2020). In other words, they are the result of adding up the average health differences between 25 and 55 year olds, men and women, and so on. Nonetheless, it is important to test for multiplicative intersectional effects by including an interaction term in case any are present; otherwise, erroneous conclusions can result (Bowleg and Bauer 2016). Does a multiplicative interaction tell us about any important policy-relevant differences? Arguably, multiplicative effects suggest that remedial policies that only focus on one type of discrimination at a time will not be fully effective for those who experience multiple types. (Although whether such a conclusion is warranted also depends on whether causality has been carefully considered to rule out alternative explanations, as discussed below.)

### Multilevel analysis of individual heterogeneity and discriminatory accuracy (MAIHDA)

One key limitation with using interaction terms is that it is not feasible to specify two-, three-, or four-way terms to take account of all possible interactions between multiple social characteristics, due to problems with ‘scalability, model parsimony, reduced sample size in some intersectional identity groups, and interpretability’ (Green et al. 2017). A potential salve to the issue of analysing multiway intersectional effects is the introduction of MAIHDA (Multilevel Analysis of Individual Heterogeneity and Discriminatory Accuracy), which arguably represented a new turn in the quantitative intersectionality literature (Evans et al. 2018; Green et al. 2017; Jones et al. 2016). MAIHDA has been labelled as the new ‘gold standard’ for investigating health disparities (Merlo 2018). It is not without criticism however, and interested readers might like to consult the recent exchange between Lizotte et al. (2019), who dispute the interpretation of MAIHDA models with regard to what they tell us about intersectional disadvantage/advantage, and the reply by Evans et al. (2019) who argue these concerns are unfounded. We agree that MAIHDA models require careful interpretation, but also that they have a number of clear advantages, which we now summarise.

The innovation with MAIHDA was to use multilevel models to nest individuals (level one) within their intersectional positions/identities (level two). Unlike conventional approaches involving interaction terms, the multilevel method can handle small intersectional subgroups due to statistical shrinkage inherent in the model, which causes smaller, more unreliable intersections to be ‘shrunk’ closer to the grand mean, and so is crucial when working with a high number of granular subgroups (Bell et al. 2019). It also partly corrects for the issue of multiple testing (Bell et al. 2019). Conceptually, the model takes intersectional subgroups as its unit of analysis and thus allows for socio-demographically ‘mapping out’ granular inequality. Further, it allows for estimating the extent to which the variance in an outcome is explained by differences between intersections versus differences within them via the variance partition coefficient (VPC). A high VPC (in a null model, without main effects included) suggests that intersections vary substantially with respect to a given outcome and that within each intersection, individuals are similar, whereas a low VPC suggests high intersectional overlap: low mean intersectional differences and high individual variance (Merlo 2018). It thus allows an understanding of the extent to which intersectionality matters *overall*, as well as the effects of *specific* intersections. With regard to finding specific intersectional effects, MAIHDA is inherently exploratory; however, it can be extended to test for specific hypotheses, for instance testing for the significance of a single, specific interaction that is hypothesised prior to modelling.

MAIHDA is also able to estimate the extent to which intersectional effects—both for each intersection and for intersectional inequality across the whole sample—are multiplicative (synergistic) or additive (layered) in their statistical constitution. This approach might find for example that there are multiplicative effects in the level of health for a particular intersection such as a working-class 55-year-old Black woman but not for the equivalent male intersection. The VPC given by MAIHDA models when the main effects are included in the fixed part of the model is a measure of overall multiplicative intersectional variation. This might be informative in showing how some outcomes are driven by multiplicative effects more than others. One potential reason for this might be that some health conditions are more visible (e.g. obesity) and therefore more subject to stigma and discrimination according to multiple social characteristics. Multiplicativity might also be context dependent, depending on the nature of discrimination in a particular society or historical period. For example, Warren (2018) observes shifts in the visual stereotypes of older women over time. As with the interaction term approach, multiplicative intersectionality might imply that particular policy interventions are needed to tackle inequality and its determinants.

### Discrimination and intersectional health

Given its social justice origins, intersectionality most naturally aligns with explanations of health inequalities based on discrimination. Intersectional differences are associated with unequal ageing because the power, resources, and life chances associated with intersectional positions/identities are important social determinants of health (Marmot et al. 2020). As Krieger (2014) notes, studies on the relationship between institutional/societal levels of discrimination and health are scant compared with those focussing on interpersonal discrimination, which are limited by under-reporting and inherent subjectivity. Studies on this relationship incorporating an intersectionality framework are rarer still, suggesting a series of urgent research questions around ‘how institutions shape individuals' positioning and experience of health’ (Gkiouleka et al. 2018).

We summarise initial guidelines in Table 1 on how to use intersectionality to research inequality.

**Table 1:** Initial guidelines for using intersectionality in research on inequality

Topic	Description	Guidelines and key issues
Defining intersections	Researchers need to decide which axes of inequality are included, how categories within each axis will be defined, and which resulting intersectional subgroups to focus on	Gender, ethnicity, socio-economic position, and age are fundamental dimensions of inequality and should be considered as a minimum. Efforts should be made to include further dimensions such as nationality and sexuality given gaps in knowledge in relation to these inequalities Taken-for-granted categorisations such as male/female and White/non-White should be questioned given their potential exclusion of marginalised people Two approaches are (i) mapping advantage/disadvantage across multiple axes of inequality and (ii) focussing on more specific intersections, informed by existing knowledge/theory. Both are justified depending on the topic/aims Too much complexity loses sight of commonalities between people and may weaken the basis for collective action
Individual heterogeneity	Even within tightly defined intersections, substantial individual heterogeneity will remain, in terms of unequal ageing/health outcomes or otherwise	It is important to distinguish between individual prediction and population level patterns; belonging to an intersectional position does not define or determine individual experience. Rather, intersectional patterning is shaped by societal factors, which can generate evidence for inequity policy People have agency and resilience to resist structural power and sources of oppression acting on their intersectional position/identity An intersectional perspective can itself inadvertently lead to stereotyping if heterogeneity is not kept in mind Intracategorical approaches, e.g. using qualitative methods are well-suited to exploring the richness of individual experience within intersectional subgroups
Use of interaction terms	Interaction terms are the main way in which researchers have so far analysed intercategorical intersectionality	Interaction terms should be specified to account for multiplicative intersectionality; otherwise, erroneous subgroup comparisons can result However, the absence of a significant interaction term does not falsify intersectionality, as it is above all a framework to understand heterogeneity and social power rather than a hypothesis Interaction terms can be specified in a range of models, but their interpretation varies for linear versus nonlinear models
Multilevel analysis of individual heterogeneity and discriminatory accuracy (MAIHDA)	An approach to analysing intersectionality specifically focussed on subgroup inequalities and heterogeneity	MAIHDA distinguishes individual versus intersectional variation, i.e. the extent to which intersections discriminate between individuals It is an alternative approach to specifying interaction terms, focussing instead on granular subgroup differences It is so far limited to cross-sectional analyses; uses age categories It can handle small or empty intersectional cells, which are problematic when using interaction terms It can distinguish additive/multiplicative effects both for individual intersections and across the whole sample. Careful interpretation of these effects is required Existing studies provide syntax examples for most popular software Inherently exploratory with regard to finding specific intersectional effects. Replication is crucial

## The life course

It is now axiomatic that the life course is central to the study of old age. If we want to fully understand later life, we must situate it within the socially constructed nature of the life course, in which social risks and socio-economic resources are highly unevenly distributed and where these structural inequalities play important roles in shaping the lived experience of old age, for example in terms of health status and income (Walker 2009). This is not to argue that the life course predetermines later life but that it undeniably exerts a powerful influence. This perspective contradicts the view of old age as a distinct phase of life and, especially, one that is ‘disengaged’ from the preceding life course (Cumming and Henry 1961). Not surprisingly early life course analyses bore the imprint of such functionalism in, for example, emphasising the role of social norms in shaping behaviour and over-emphasising the power of individual choice (Clausen 1986; Elder 1975; Neugarten et al. 1965). While not underestimating the pioneering nature of these early developmental approaches, especially Elder’s, their primary purpose was not to attempt theoretical insight but, rather, the life course was utilised as a conceptual framework for conducting research and interpreting data concerning adult role transitions. Focus on the institutionalisation of the life course by sociologists like Dannefer (1987) and Kohli (1986) went hand in hand with the political economy perspective in gerontology, which brought the life course to centre stage in the study of old age. Social gerontologists working within this structural perspective analysed life course inequalities deriving from the work course and SEP (Guillemard 1986; Walker 1981), gender (Arber and Ginn 1991; Estes 2006) and race and ethnicity (Blakemore and Boneham 1994; Nazroo 2003). Recently, the combination of biogerontological and sociological analyses, for example in the New Dynamics of Ageing Programme (https://www.newdynamics.group.shef.ac.uk/), has led to the introduction of a radical public health perspective, which places a particularly strong emphasis on the life course influences on unequal ageing—for example in tracing associations between childhood deprivation and late life functioning (Foster and Walker 2015; Kuh et al. 2014; Walker 2018). Thus, a life course lens forces us to take a dynamic view of ageing rather than a static one of old age (Arber and Evandrou 1993). This dynamism has yielded some fruitful research avenues, including the notion of cumulative advantage/disadvantage—the systematic tendency towards divergence in socio-economic resources and status over time (Dannefer 2003).

## Mutual synergies between intersectionality and the life course

Dressel et al. (1997) provided an early outline for how intersectionality could help frame a more inclusive and critical gerontology, to better understand ‘the dynamic interplay of race, class, gender, and ageing’ (1997: 579). They argued that systems of discrimination based on age, gender, ethnicity, and social class intersect across the life course to lead to varied older age outcomes, causing us to rethink taken-for-granted concepts such as retirement, adjustment to old age, and healthy ageing. For example, a study of African American grandmothers suggested that age was not seen as a prominent social identity in the face of lifelong experiences of poverty, racism, and sexism (Dressel and Barnhill 1994). Similarly, among low-income African Americans where redundancy and unemployment are common, the concept of voluntary retirement may have little meaning. Caregiving is a further area where an intersectional framework is illuminating (Dressel et al. 1997). Government policies commonly devalue both paid and unpaid caregiving—as illustrated starkly by the Covid-19 pandemic. Furthermore, ethnic minority women are concentrated in the lowest paid jobs and are particularly disadvantaged in terms of older age income if they are forced to leave work to care for someone. Finally, Dressel et al. (1997) also discussed intersectional effects in patterns of inequality over the life course, now well-known in gerontology: age as leveller, persistent inequality, and finally cumulative disadvantage/advantage. More recently, from an ethnographic perspective, Ferrer et al. (2017) outline four elements of an ‘intersectional life course perspective’: life events, timing and structural forces, local and globally linked lives, identities and categories of difference, and agency, domination, and resistance. They argue for examining the interconnections between life events, transitions, trajectories, and systems of domination over the life course.

These existing attempts at combining intersectionality and life course perspectives provide the point of departure for the present analysis, incorporating recent methodological developments, and focussing on unequal ageing with a particular emphasis on health.

### The life course, intersectionality, and categorisation

From a life course perspective which emphasises the dynamic and temporal nature of social categories, gender, ethnicity, and SEP and their disparities are both causes and consequences of social stratification (Richardson and Brown 2016). At the same time, the overarching point of intersectionality, that axes of social inequality are differentiated according to others, can help to strengthen a focus on diversity in gerontology.

Seldom acknowledged in the intersectionality literature, yet fundamental to life course dynamics, is the view of the individual life cycle as comprised of various socially constructed life stages and role transitions: ‘Lives change as relationships and social roles change’ (Elder and Giele 2009: 9). The ‘traditional’ life cycle describes how people transition from childhood to independence, marriage, childbearing, and ‘growing old’, and with respect to productive roles, from education to employment and then retirement. The ‘age-graded normative organization of society’ governs when key transitions such as marriage or retirement are considered normal—the so-called ‘institutionalisation of the life course’ (Kohli 1986). Norms dictate the ‘appropriate’ time for life transitions and people can be early, late, or on time and are generally aware of their timing (Elder 1975).

From an intersectionality perspective, role transitions are a fundamental part of development and social identity (Vespa 2009) and also entail moving through different structural positions in the social hierarchy, with differential access to power and resources. Norms around timing are intersectionally patterned, as with Dressel et al.'s (1997) example of deprived ethnic groups and retirement. With respect to intersectional categories and time, people usually belong to a certain gender and ethnic group over the lifespan, such as the category of ‘Black woman’. As Black women (or any other gender/ethnic group) age, they transition through different social and re/productive roles and occupy different structural positions. Social identity and lived experience therefore constantly evolve over the lifespan (Ferrer et al. 2017). Intersectional patterning in how people move through life transitions can help to explain intersectional outcomes. For example, Aisenbrey and Fasang (2018: 1) use sequence analysis to examine intersectional differences in work and family life courses and career outcomes, finding for example that ‘for Black men high prestige careers are only accessible if they are in stable relationships with maximum one child’. This example compellingly shows how social ties to significant others ‘establish forms of socialization and control in channelling individual actions and decisions’ (Elder and Giele 2009: 10). It also demonstrates how people move between SEPs over the life course, which is also the case with other positions/identities typically used in intersectionality research, e.g. based on disability, migrant status, or neighbourhood. An understanding of transitions, both in terms of social categories and how these are tied into social roles transitions, offers a much-needed rich and dynamic view of intersectional subgroups.

The existence of social categories also depends on historical time. For example, contemporary societies exhibit substantial ethnic diversity (Salway et al. 2020), and traditional patterns of retirement have become increasingly fragmented in contemporary society, now characterised by bridge jobs, flexible retirement, and unretirement (De Tavernier et al. 2019). The meaning of social categories such as ‘Black’ depends on context. For example, it means something different to be Black in America compared with the UK and in 2020 compared with 1920. The dynamic nature of categorisation means that exposure to the intersectional ‘matrix of domination’ is not monolithic but, rather, people belong to multiple social categories over the life course and the meaning of this belonging depends on historical time (Ferrer et al. 2017). This insight allows for conceptual separation of age and cohort effects. For example, Elder (1974) showed how the Great Depression was a formative experience that shaped particular views of and orientations towards the world for the cohorts that experienced it (Gubrium and Holstein 2006). Often, in cross-sectional intersectionality research age is split into 5- or 10-year bands, yet this treatment sidesteps the importance of life course dynamics. So far the distinction between age and cohort has received little acknowledgement in intersectionality research (with some exceptions such as Warner and Brown 2011). Importantly, the same age can have a different meaning for those in different intersections given intersectional variation in role transitions. However, to properly unpack these dynamics, longitudinal approaches are needed, as we explore below.

Alongside a concern with historical time, both intersectionality and life course researchers also often emphasise the importance of spatial context (Elder and Giele 2009; Scott and Siltanen 2016). Recently, geographers have begun to consider the crossover with intersectionality (Hopkins 2019), though methods for how to investigate spatial intersectionality are still being established. Spatial context by contrast has been well-examined in gerontology in how it shapes social roles and identities including in different societies, regions, and localities, or as with early life course analysis of childhood development, schools, neighbourhoods, and communities (Elder and Giele 2009). For example, in deprived neighbourhoods, the role of age may be less salient, and in certain countries disability is more common than in others. Dressel et al. (1997: 593) elaborate, asking:

in declining neighbourhoods where work is disappearing, what is the role of elders in terms of their contribution to the socialization of children and adolescents into normative work roles? Do such roles vary by ethnicity, gender, or other factors?

They note that answers to these questions have the potential to ‘link issues of individual, group, and community well-being in ways that could shape more effective social policies and community development activities’. Similarly Ferrer et al. (2017) suggest that people formulate their identities based on relationships with multiple family members and multiple generations, and these relationships taken place across transnational spaces. The recent turn to localism in public health in the UK, which devolved responsibility to city councils (Phillips and Green 2015), suggests a role for a local intersectional approach to generate knowledge useful for informing locally relevant inequity policy.

### The life course, intersectionality, and agency

As noted, intersectionality scholars are keen to stress the strengths and assets of those who experience oppression and how they can resist it, how social identities are fluid and multifaceted, and how the expression of agency is dependent on time and place. Similarly, in the life course perspective, it is generally accepted that people have a degree of control over their roles and situations, constructing ‘their own life course within given constraints’ (Elder and Giele 2009; Walker 2006). However, agency remains the subject of active debates in the field, for example concerning choice, constraints, action, and resilience (Gilleard and Higgs 2010; Marshall and Clarke 2010). Clausen’s (1991) notion of ‘planful competence’—a putatively stable personality characteristic or individual strategy formed by adolescence and a predictor of life course success (Dannefer 2018) highlights the interplay with social structure over the lifespan. As noted by Dannefer and Miklowski (2006), agency can only be expressed within institutional structures, language being the most fundamental example. The way in which particular uses of language exclude certain groups and limit life chances is well-documented, classically in relation to social class and education (Bernstein 1964). These dynamics may help to explain the ways in which intersectional position and identity become separated under certain social contexts (Bauer 2014). People may forefront different identities in different social contexts or at different points in their life course (Kapilashrami et al. 2015).

Dannefer and Miklowski (2006) argue that the concept of social risk helps us to understand the role of agency in late modern societies. Key risks once stemmed from a lack of information to inform action, whereas now there is a risk of *too much* information, placing more responsibility for action on the individual. One example is the UK pension system. Not only is it seen as one of the most complex in the world (Pensions Commission 2004), but it has also been shifted towards individual responsibility with the introduction of a self-managed defined contribution system. From an intersectionality perspective, social risk is not only shifted to individuals, but to intersecting deprivations because these structure financial acumen (Holman et al. 2018). As well as language, knowledge, and other dispositional structures, and the ways in which they can act as vehicles of discrimination, intersectional differences may also structure the ways in which people are enabled or constrained in expressing agency as they shape access to power and resources.

### The life course, intersectionality, and patterns of inequality

Population level life course analysis examines patterns of inequality over the life course, focussing on birth cohorts or other subpopulations (Dannefer and Kelley-Moore 2009). Observed patterns suggest that population processes or characteristics are influenced by social, political, or economic structures and policies (Dannefer and Kelley-Moore 2009). Such factors explain both differences between cohorts and stratification within them. Methodologically, trajectories are used to understand how outcomes such as health or income change as people age individually, and whether this change is associated with a characteristic or experience that varies between people. George (2009) points out that social scientists are used to thinking in terms of between person differences, such as how those in SEP are associated with differences in health. Trajectory analysis extends this by allowing for a consideration of how *changes* in SEP over the life course influence health. In this, a number of potential dynamics emerge, such as time spent in certain SEPs (duration) and at what age (timing/critical periods), how the order of SEP statuses might influence health (sequential effects), or how certain SEP transitions might constitute a big life change (turning points).

The extension to multiple social characteristics and their intersections is a natural progression of such analyses. Rather than examining only SEP, trajectory analysis might consider timing, order, and turning points with respect to intersectional subgroups, constituting an ‘intersectional trajectory’ approach. Dressel et al. (1997) discuss the potential for intersectional effects in three patterns of inequality over the life course, now well-known in gerontology: age as leveller, whereby gender, ethnicity, or class inequalities are gradually washed away by the strength of age effects in later life; persistent inequality, whereby inequalities persist over the lifespan; and finally cumulative disadvantage/advantage, the notion that early disadvantages/advantages amplify and widen over the lifespan (Dannefer 2003).

The cumulative disadvantage/advantage hypothesis suggests that early life inequalities in disadvantage/advantage set the scene for (though do not determine) further disadvantage/advantages and therefore that childhood circumstances and experiences have particularly formative and cumulative effects (O’Rand 2009). With respect to health Ferraro and Kelley-Moore (2003) found that obesity during adulthood, but not childhood, follows this pattern. They argue for a focus on compensatory mechanisms, both in terms of exiting the risk state of being obese, but also countervailing mechanisms including exercise, which have differential effects. From an intersectionality perspective, researchers might examine the extent to which intersectional position/identity at a particular stage of the life course is contingent upon previous stages (as with the aforementioned research by Aisenbrey and Fasang (2018)) and the role of compensatory mechanisms in breaking chains of disadvantage/advantage. For example, the relationship between childhood intersectional position and markers of disadvantage related to early conditions and experiences, such as child abuse, changes in family composition including parental death or divorce, and health during childhood or adolescence might be considered. Ferraro et al. (2016) argue that researchers have so far mostly considered these factors in isolation, and instead we need to think about how such domains of disadvantage are related to each other, e.g. in having simultaneous, overlapping, or sequential effects, echoing the same underlying logic of intersectionality. Similarly, as with an intersectionality perspective, advantages are in theory as pertinent as disadvantages, such as positive childhood experiences of parental warmth, household security, or the availability of books in the household (Brunello et al. 2017).

Doren and Yin (2019) examine intersectional life course trajectories in the gender gap in earnings and find that the gap increases most with age for whites and those with college education, such that male advantage is most pronounced for those who also have racial and ethnic advantages. Some analyses with health as an outcome have been carried out (Ailshire and House 2011; Brown et al. 2016; Richardson and Brown 2016; Warner and Brown 2011). For example, Richardson and Brown (2016) found a multiplicative effect of ethnicity and gender on hypertension risk trajectories. However, these analyses have so far focussed on the later life course and there is need to also consider earlier life conditions. Further, these studies use interaction terms to examine intersectional effects in trajectories, which is a valid, though as noted a somewhat limited approach. There is therefore a methodological and conceptual gap between recent advances in intersectionality using the MAIHDA approach (Merlo 2018) and trajectory analysis. An extension to the MAIHDA method would potentially allow for partitioning variance between individuals and intersectional groups, adding a third level to model change. Such an approach would speak to current gaps in understanding in both life course and intersectionality scholarship, but would push the limits of typical sample sizes at least in longitudinal surveys.

### The life course, intersectionality and discrimination

Intersectionality focuses on discrimination in explaining inequality as it is concerned with social differences which entail relationships of power, privilege, and oppression. However subgroup inequalities not only result from discrimination, but also other social, psychological, or even physiological or genetic differences. One challenge with intersectionality research is therefore to attribute intersectional differences to wider systems of discrimination and their interconnections (the ‘matrix of domination’ Collins 2002).

Research in this area might proceed by showing that intersectional discrimination has a life course dimension. People are simultaneously affected in different ways by multiple overlapping policies depending on their intersectional position, which Beckfield et al. (2015) describe as ‘institutional imbrication’. For example, age, cohort, and gender influence who is affected by rising state pension ages, and we might expect that the ability to respond to these changes is further differentiated by ethnicity and SEP. Policies and institutions are therefore a key way in which the ‘matrix of domination’ influences intersectional position. In addition, successfully navigating institutions is often associated with key life transitions, such as entry into certain schools, universities, professions or countries, and institutional discrimination therefore potentially leads to further exclusion and marginalisation. In this way, institutions mediate one’s intersectional trajectory. For example, organisational narratives help to construct identities of students, employees, or care home residents as having potential and being worthy of investment, or as hopeless and ‘dead wood’ (Dannefer 2018). On the social psychological level, Bengtson’s (1973) concept of ‘induced incompetence’ explains how vulnerable individuals—originally on the basis of age but also on other axes of inequality—are susceptible to being labelled as incompetent.

We might expect that duration, timing/critical periods, and sequential effects associated with intersectional positions/identities will have particular consequences for ageing outcomes. For example, a study by Bécares and Zhang (2018) found that accumulated interpersonal discrimination has a negative impact on the mental health of older ethnic minority women. Timing of discrimination also matters, for example, the younger the age of first arrest the greater the risk of subsequent incarceration (Elder and Giele 2009), and racial bias in arrest is well known. From a health development perspective, stress and social adversity are embedded into the biology of human development during sensitive and critical periods, when ‘biological and behavioural regulatory systems are being initialized’ (Halfon et al. 2014). Marginalisation, exclusion, and discrimination, as causes of social stress (Masters et al. 2012), are therefore important mechanisms through which the matrix of domination becomes biologically embedded in intersectional identities.

A further factor to consider in a life course perspective is that the ‘matrix of domination’ changes over historical time, and people therefore experience sexism, age, racism, etc., differently in different historical periods. Examination of trends in ageism in particular has been identified as a research gap (Palmore 2015). One notable exception is the work of Twigg (2013) who observes a shift in ageist stereotypes from physical limitations to cosmetic appearance, with a particularly severe impact on older women. Health or social policy can help mitigate the health effects of discrimination in different historical periods. For example, as Halfon et al. (2014: 348) note ‘the experiences of low socio-economic status, discrimination, and racial segregation could have different effects on health for different cohorts based on compensatory and mediating factors such as the availability of healthcare, or the impact of different social policies’.

Corna (2013) suggests how policy contexts including work and family experiences, pension structure, and tax policies can be incorporated in empirical work. First, when it is not possible to link contextual information with microlevel data, the former can be used to interpret findings on the latter. For example, this might be an examination of national policies on social care, pensions, and retirement and how they might discriminate different groups. More widely, policy contexts such as austerity (Greer Murphy 2017), neoliberalism (Bell and Green 2016), and corporate/commercial interests (McKee and Stuckler 2018) shape intersectional inequalities. Second, contextual information can indeed often be linked to micro data, e.g. survey or census data, to facilitate a cross-national perspective (or indeed other area-level analyses). Such comparisons are illuminating in that racism, sexism, ageism, and so on are historically and contextually specific (Veenstra 2011). Thirdly, cross-national panel data would also allow for incorporating a dynamic understanding of how different social policy contexts influence intersectional outcomes. Multilevel longitudinal analysis is well suited to such an approach as it is able to incorporate multiple levels representing different contextual factors. To extend Corna’s list, methods for causal inference might also be used, such as natural experiments or the use of instrumental variables, to show that social policies have differential intersectional effects.

Empirically, MAIHDA may provide a tool to investigate the role of the wider context in shaping the total amount of intersectional variation in health, whether this is multiplicatively driven, and how this plays out within individual intersections (Merlo 2018). Different configurations of social and political determinants of health, or different matrices of domination, should be seen as central to the wider context. Yet given their socio-historical contingency, a historical perspective is needed to correctly position the influence of sociological factors on population health.

Table 2 summarises areas of synthesis between intersectionality and life course analyses and how they can be used to understand unequal ageing.

**Table 2:** Synthesising intersectionality and life course analyses to understand unequal ageing

Areas of synergy	Description	Examples and potential approaches
People change intersectional subgroups over the life cycle and could therefore be said to follow an ‘intersectional trajectory’	People occupy a series of structural positions/social identities over the life cycle Social axes of inequality are both causes and consequences of social stratification Gender and ethnicity are relatively stable characteristics, while SEP changes over the life cycle Intersectional trajectories might be consistent with age as leveller, persistent inequality, or cumulative disadvantage/advantage patterns of unequal ageing Social roles in relation to reproduction and production, as well as personal relationships, are intertwined with intersectional trajectory Social roles and relationships channel individual actions and decisions The norms and meanings regarding key roles and transitions may be intersectionally patterned	Examine how people navigate role transitions and intersectional patterning in this Analyse ethnicity by gender outcomes depending on time spent in certain SEPs (duration) at what age (timing/critical periods), how the order of SEP statuses might influence health (sequential effects), or how certain SEP transitions might constitute a big life change (turning points) Aisenbrey and Fasang (2018) find that high prestige careers are mainly only accessible for Black men if they are in stable relationships with a maximum of one child Dressel et al. (1997) highlight how retirement might have little meaning among low income African Americans Richardson and Brown (2016) find a multiplicative effect of ethnicity and gender on hypertension risk trajectories
People employ agency to resist discrimination and shape their own identities across the life cycle, within given constraints	Resource constraints and institutional structures limit the possibilities for agency Risks in relation to knowledge and information, e.g. in relation to the pension system may be intersectionally patterned People actively shape their positions/identities over time based on their age, gender, ethnicity, class, and other characteristics	Holman et al. (2018) suggest women disadvantaged according to SEP and ethnicity were less able to mitigate the damaging effects of increases to the women’s state pension age because they were less likely to be aware of the change and less likely to have the financial knowledge to make complex pensions decisions Walker and Naegele (1999) showed the variations in political participation between different groups of older people, based on SEP, in different EU countries
Intersectional patterning and its significance for unequal ageing varies by historical time and spatial context	Different schools, neighbourhoods, regions, or countries have different intersectional diversity, which changes over time (e.g. changing numbers of professional ethnic minority women) Intersectional subgroups take on different meanings in different contexts. It means something different to be a working-class 55-year-old Black woman in 1920 versus 2020, and in the UK versus the USA Intersectional patterning may have more explanatory power with respect to unequal ageing in certain historical times and spatial contexts than in others From an intersectionality perspective, discriminatory norms, policies, and institutions are key explanations for why intersectional outcomes vary by context (see below)	Examine time/place differences in intersectional diversity Conduct MAIHDA analysis to examine within and between intersectional variation in different historical times and spatial locations to understand explanatory power of intersectional patterning Examine the ageing of different cohorts of disabled people and people with intellectual disabilities Compare relevance of area deprivation versus individual SEP in explaining health inequalities, and how this varies by age, gender, and other axes of inequality Examine the relevance of age in deprived neighbourhoods or deprivation in neighbourhoods with a high average age Conduct intersectional analyses on a local or regional scale to generate place-specific evidence Informal care role of older adults may change in neighbourhoods with high unemployment (Dressel et al. 1997)
People are affected by multiple forms of discrimination over the life cycle and according to historical time and spatial context	The impact of discrimination on unequal ageing depends on life course dynamics, e.g. duration, timing/critical periods, sequential effects Sustained discrimination over the life cycle on the basis of characteristics other than age influences how later life age discrimination is experienced Meso-level discriminatory mechanisms, e.g. labelling of individual potential, and the social-psychological dynamics of internalised incompetence reproduce inequalities over the life cycle Individuals experience differential ‘institutional imbrication’, i.e. exposure to multiple policies/institutions/stereotypes not only based on their intersectional position/identity, but on their country, age, and cohort Experiences of discrimination varies by time and place depending on the prevalent ‘matrix of domination’ (see below)	Analyse inequalities in life expectancy and healthy life expectancy according to age, cohort, gender, race, and ethnicity Examine differences in reported interpersonal discrimination across time and place Examine how individuals experience multiple forms of discrimination across different contexts, e.g. ageism in one policy and sexism in another, and multiplicative forms of discrimination, e.g. some policies are both sexist and ageist Policy contexts can be used to interpret individual outcomes, can be directly linked to individual data, or cross-national panel data can be used for a comparative perspective of changing policy contexts over time Bécares and Zhang (2018) found that accumulated interpersonal discrimination has a negative impact on the mental health of older ethnic minority women Dressel and Barnhill (1994) found that for African American grandmothers age was not seen as a prominent social identity in the face of lifelong experiences of poverty, racism, and sexism
Ageism, sexism, racism, and other forms of discrimination and their interconnections (the ‘matrix of domination’) vary by historical time and spatial context	Social policies and institutional practices, such as the welfare state, education, immigration, social care, and retirement are more or less discriminatory depending on historical time and spatial context Such policies and practices can discriminate on the basis of single or multiple social characteristics The wider socio-political context, e.g. austerity and neoliberalism shapes discrimination and oppression The nature and prevalence of stereotypes changes over time	Policy analysis of how policies discriminate based on both single and multiple axes of inequality at a time, e.g. ageism and stereotypes of older men versus women The transformation from ‘worn out’ older workers of the early twentieth century to the ‘productive ageing’ of the early twenty-first century (Macnicol 2006) Shifts in the visual stereotypes of older women over time (Warren 2018) The cultural turn in ageist stereotypes from physical limitations to cosmetic appearance, with a particularly severe impact on older women (Twigg 2013)

## Conclusion

This paper has argued for a conceptual dialogue between the frameworks of intersectionality and the life course. These analyses are preliminary owing to the many unanswered questions that arise from beginning this endeavour. It is now widely accepted that a life course perspective is essential to understanding unequal ageing, with health being a fundamental dimension, at both individual and societal levels. Equally, it is essential for gerontological students to understand that ageing is unequal with respect to a number of intersecting axes of inequality which operate simultaneously and often in combination. There is a strong case, therefore, for moving towards the integration of both perspectives, based on a dialogue with mutual benefits. We have demonstrated that there are clear synergies and great scope for mutual enrichment between intersectional and life course perspectives, particularly regarding categorisation, agency, patterns of inequality and discrimination. These synergies hold exciting opportunities to bring new insights to unequal ageing and its attendant health inequalities.
